# Failure to replicate effects of parent‐delivered early language intervention: Evidence from a randomised controlled trial with implications for universal language intervention

**DOI:** 10.1002/jcv2.70064

**Published:** 2025-11-06

**Authors:** Kelly Burgoyne, Laura Boundy, Paivi Eerola, Qing Zhang, Jochen Einbeck, Helen Cramman, Vic Menzies

**Affiliations:** ^1^ Manchester Institute of Education University of Manchester Manchester UK; ^2^ School of Education Durham University Durham UK; ^3^ Great Ormond Street Institute of Child Health University College London London UK; ^4^ Department of Mathematical Sciences Durham University Durham UK

**Keywords:** early intervention, education, language, parents, RCT design, school readiness

## Abstract

**Background:**

Parent‐led, preschool language interventions may help to mitigate the risk of poor language and literacy outcomes associated with lower socioeconomic status. This study builds on two previous evaluations of a parent‐led early language teaching programme, which demonstrated mixed findings.

**Methods:**

A two‐armed randomised controlled trial was conducted with 372 families and their children aged 3–4 years, recruited from 43 schools across the North‐West of England. Families were randomly allocated to either the programme group (*N* *=* 186) who delivered the 20‐min programme 5 days a week for 30 weeks, or the control group (*N* *=* 186) who received storybooks at the end of nursery. Language and early literacy outcomes were assessed at baseline (*t*1), immediately after the 30‐week programme (*t*2) and 10 months later (*t*3).

**Results:**

Families delivered on average 65% (20/30 weeks) of the intended sessions. There were no significant group differences in language outcomes at immediate (Hedges' *g* = −0.05 to 0.11) or delayed post‐test (*g* = −0.13 to 0.10). Further, no significant group differences were found on measures of early literacy (*g* = −0.11 to 0.12), the Home Learning Environment (*g* = −0.07) and School Readiness (*g* = −0.15). Effects of the programme on language skills were stronger at higher dosage (*g* = 0.33 for those completing >90% sessions) and for those with weaker language skills.

**Conclusions:**

Findings showed no significant effects of intervention. Reasons for null findings are discussed with implications for universal interventions. Recommendations for future trials evaluating interventions of this nature, including targeting of families and children most in need of support, are discussed.

**Trial Registration:**

This trial was pre‐registered with the ISRCTN registry [ISRCTN52533968 https://doi.org/10.1186/ISRCTN52533968] on 13/07/2022.

## Key Points

**What's known?**

- Evidence of a positive impact on child outcomes following parent‐delivered teaching programmes is inconsistent.

**What's new?**

- A large scale, pre‐registered, randomised controlled trial of a parent delivered early language teaching programme showed no significant effects on child language, school readiness, or the home learning environment.
- Evidence indicated potential effects downstream, particularly for children with the weakest language skills at the start of teaching and those receiving more teaching.

**What's relevant?**

- Findings argue for careful targeting of parent‐delivered intervention programmes, focussing resources on disadvantaged families and children with the greatest learning needs.

## INTRODUCTION

Oral language skills are foundational for academic success, particularly early literacy (Hulme et al., 2015; Sloat et al., 2015), numeracy (Purpura et al., 2017), and socio‐emotional development (Clegg et al., 2015). Consequently, whilst language development is highly variable over time leading to low levels of individual prediction (Armstrong et al., 2017; Dale et al., 2003), children who start school with limited language and vocabulary skills are at risk of poor academic outcomes (Melhuish et al., 2013; Roulstone et al., 2011). This disadvantage persists beyond the school years affecting health, social, and economic outcomes later in life (Eadie et al., 2021; Fitzpatrick et al., 2020).

Differences in early language skills are linked to both socio‐economic status (SES) and the home learning environment (HLE) (Marmot, 2020; Sammons et al., 2015). Indeed, aspects of the HLE including parent‐child interactions and resources available in the home are more influential for early learning than SES (Aikens & Barbarin, 2008) and may mediate SES and learning outcomes (Niklas & Schneider, 2013). A key context where high‐quality interactions can occur is shared book reading and research has highlighted a positive association between parent‐child reading and language outcomes (Leech et al., 2022; Mckean et al., 2015; Murray & Egan, 2014; Murray et al., 2023).

For some families, particularly those who are socially disadvantaged (Le Roux, 2012), factors including limited access to books and resources (Roulstone et al., 2011) and a less interactive style of book reading (Mol et al., 2008) often result in fewer language learning opportunities via shared reading at home. These social disparities have been exacerbated through austerity cuts to Children's Centres (Sammons et al., 2023), and more recently through closures of early education settings during the COVID‐19 pandemic (Bowyer‐Crane et al., 2021): Emerging evidence points to a negative impact on early language development for low‐SES children for whom interactions with early education practitioners provide important enrichment of language input received at home (Davies et al., 2021). Therefore, there is a growing need to establish effective language interventions targeting children before they start school.

The link between shared book reading and language and literacy outcomes makes it a promising vehicle for language intervention (Fricke et al., 2017; Mol et al., 2008). A recent meta‐analysis of 19 RCTs found that dialogic reading interventions had significant effects on expressive and receptive vocabulary (effect sizes *d* = 0.41 and *d* = 0.26; Dowdall et al., 2020). However, smaller effects (*g* = 0.19) on early language, which were short lived and non‐significant at follow‐up (*g* = 0.14), were reported in another meta‐analysis of shared book reading interventions (Noble et al., 2019). Both meta‐analyses highlighted that many interventions were low dose, running only a few weeks; longer‐term interventions may yield larger effects. Furthermore, dialogic reading practices alone may not be sufficient to improve language in low‐SES children (Mol et al., 2008). For these families, including more direct language instruction may be more effective at promoting oral language (Bowyer‐Crane et al., 2008; Fricke et al., 2017).

One early language intervention that incorporates these features is the Parents and Children Together (PACT) programme (Burgoyne et al., 2018). PACT supports parents to implement shared reading alongside direct teaching of vocabulary and narrative skills over 30 weeks. A randomised controlled trial (RCT) including 208 preschool children and their families recruited from Children's Centres in socially deprived areas in the UK found significant effects of the programme on children's language (*d* = 0.21) and narrative (*d* = 0.36) skills immediately following the programme. Effects on language were maintained 6 months later (*d* = 0.34) at which point children receiving the intervention also displayed better word reading (*d* = 0.35) and letter sound knowledge (*d* = 0.42).

A second trial of the programme (Burgoyne et al., 2024) failed to replicate these findings; however, this second trial was significantly impacted by the COVID‐19 pandemic making it difficult to interpret these findings. This paper reports outcomes from a third RCT of the PACT programme.

## METHOD

**FIGURE 1:**
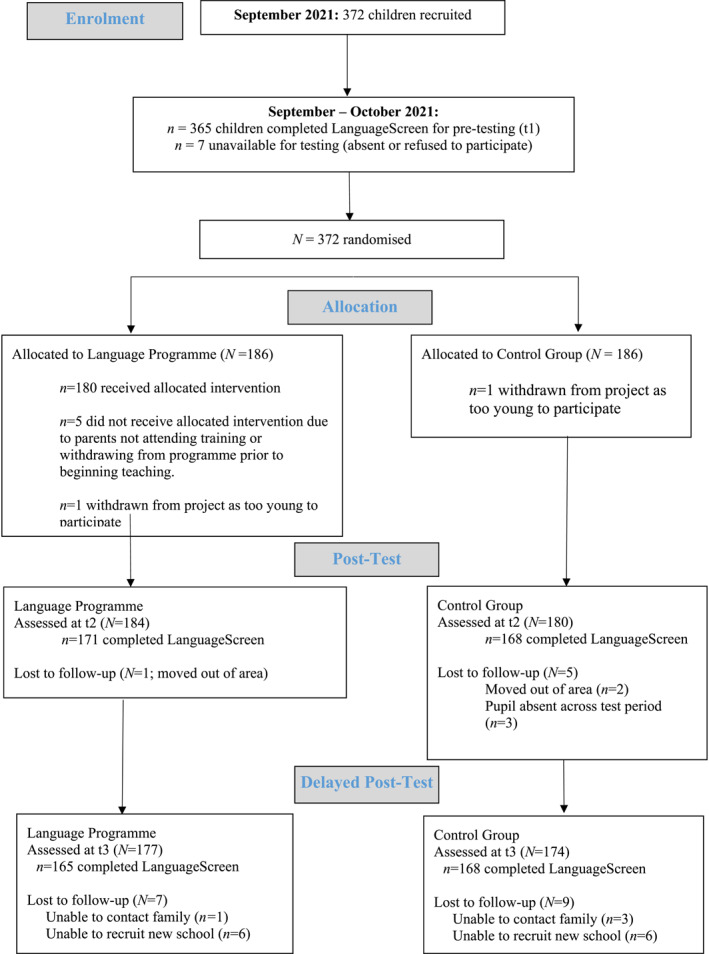
Consort diagram showing flow of participants through RCT study. RCT, randomised controlled trial.

The current trial involved 372 families from Greater Manchester and Lancashire (UK) to evaluate PACT, a parent‐delivered teaching programme targeting early language development in pre‐school children (aged 3–4 years). Ethical approval was granted by Durham University Research Ethics Committee. Informed parental consent was obtained for all children. Details of participant recruitment, allocation and flow through the study are summarised in the CONSORT diagram (Figure 1).

### Sample size

Sample size was calculated based on an assumption of 80% Power, *p* < 0.05, two‐tailed, a 0.1 intra‐school correlation, and a pre‐post test correlation of *r* = 0.60. A power calculation showed that with *N* = 186 per arm and a pre‐post test correlation of *r* = 0.60, there was better than 80% power to detect a difference between groups equivalent to *d* = 0.22 (*p* < 0.05, 2‐tailed).

### Participants

A total of 50 schools in the North‐West (UK) were initially recruited to take part. To take part, nursery schools had to be state‐funded and attached to a primary school. Schools and nurseries who participated in the second PACT trial (*N* = 80) (Burgoyne et al., 2024) were initially offered the new trial: 25 recruited settings had participated fully in the second PACT trial while four settings had only been involved in the second PACT trial at delayed post‐test (as children taking part in the study had moved to these settings in Reception year). Other recruitment methods included online information events, social media, and cascading of information through Local Authorities.

Eligibility criteria for families were (1) child in nursery (aged 3–4 years), (2) parent(s) able to read and understand English (3) no plans to move out of area. Exclusion criteria were (1) twins or siblings in the same year group, (2) child had a known or suspected developmental disorder other than developmental language disorder (e.g., autism), (3) participation in previous PACT trials. To remain in the trial, schools were asked to recruit a minimum of 4 families. Settings unable to do so (*N* = 7) were withdrawn, leaving 43 schools in the trial (Greater Manchester (17), Blackpool (5) and Lancashire (21)). Whilst these broad geographical areas score highly on indices of multiple deprivation, individual nursery postcodes indicated considerable variability across settings with as many nurseries located in areas of little‐moderate deprivation (*n* = 20) as areas of high deprivation (*n* = 23). Participating settings recruited 372 children (175 boys) in total with the number of families recruited per setting ranging between 4 and 19 (mean = 8.7).

Children were aged between 3 years, 0 months and 4 years, 1 month at pretest (mean age in years = 3.62). Parents reported that English was not the main language used at home for 20 children (5.38% of the sample; *n* = 11 in the PACT Programme group; *n* = 9 in the Comparison group). Families' socioeconomic status was based on eligibility for Early Years Pupil Premium (*N* *=* 66 at baseline) and home postcodes using the English Indices of Deprivation (2019). Where 1 = ‘most deprived’ and 10 = ‘least deprived’, 39.45% of participants lived in areas 1 or 2, with 9.45% in areas 9 or 10 (*N* *=* 372; missing data: *N* = 2). The mean average was 4.04. Further details on setting and participant sample characteristics can be found in Supporting Information S1: Tables S1 and S2.

The study is a two‐armed RCT, with children randomly allocated to the Programme Group (*N* *=* 186), where families engaged in the PACT language programme, or the Comparison Group (*N* *=* 186), where families received a box of storybooks at the end of nursery. Random allocation was at the pupil/parent level and stratified by school, which was considered the optimal design given the maximum number of schools we had capacity to deliver to and maximising the power of the trial to detect an effect. Potential contamination between groups was controlled by emphasizing the importance of group allocation, and instructing Programme Group parents and schools not to share materials with control group families; the likelihood of this was further minimised by distributing new materials (many of which were not reusable) in stages as Programme Group families progressed through the programme. Implementation and process evaluation (Menzies et al., 2024) identified minimal contamination between groups. Group allocation was conducted independently by Durham University and randomisation was balanced across treatment groups for ability to complete pretests before randomisation (at randomisation pre‐test data was complete for 357 children, 10 children completed pre‐test after randomisation and no baseline data was available for 7 children due to absences at pre‐test or the child not engaging). Following randomisation, 2 children (one intervention group, one control group) were withdrawn from the trial as they were too young to participate.

### Assessments

Children were assessed in their setting by school staff using the LanguageScreen assessment at three time points: pre‐test in September/October 2021 before randomisation (*t*1), immediate post‐test in June/July 2022 after the 30‐week intervention (*t*2) and delayed post‐test 10 months later in May/June 2023 (*t*3). At immediate (*t*2) and delayed post‐test (*t*3) children were also assessed by a researcher visiting school using a battery of language and (at *t*3) early literacy assessments. Whilst it was not possible to complete these researcher‐delivered measures at *t*1 due to ongoing COVID‐19 restrictions, these measures at *t*2 and *t*3 allowed the researchers to explore any potential bias in the school‐delivered assessments and afforded more in‐depth assessment of language skills. Schools and parents received a £10 gift voucher for each child‐assessment.

### Language measures

#### School delivered (*t*1, *t*2, *t*3)

*LanguageScreen* is a computerised, standardised language assessment app, with 4 subtests measuring expressive and receptive vocabulary, sentence repetition, and listening comprehension (https://www.languagescreen.com/). The test has high levels of reported reliability (*α* = 0.92; test‐retest *r* = 0.78) and validity (*r* = 0.74) based on a large sample of children aged 3; 06 to 8; 11 (Hulme et al., 2024). Assessment was delivered by school staff on a tablet via the app and took 10 min per child. Scoring is automated.

#### Researcher delivered (*t*2, *t*3)

These assessments were delivered by blinded researchers on a one‐to‐one basis with each child at their school. The assessments took place in a single 20‐min session per child at *t*2, and in two, 20‐min sessions at *t*3.

##### Expressive and receptive vocabulary (*t*2, *t*3)

*Receptive vocabulary* was assessed using the British Picture Vocabulary Scale‐3 (BPVS‐3) (Dunn et al., 2009) in which the child points to a picture (of 4) that represents a given word. *Expressive vocabulary* was measured using the Clinical Evaluation of Language Fundamentals Preschool 2 UK (CELF‐Preschool 2 UK) Expressive Vocabulary subtest (Semel et al., 2006) in which children are shown a picture and asked to name it. Expressive vocabulary was also assessed through the *Information Score* on the Renfrew Action Picture Test (4th Ed.) (APT; Renfrew, 2010) where children are shown a series of pictures and asked questions about them.

##### Expressive grammar (*t*2, *t*3)

The *Grammar Score* from the APT provided a measure of expressive grammar.

##### Early literacy skills (*t*3)

Subtests from the York Assessment of Reading for Comprehension (YARC; Hulme et al., 2009) measured letter‐sound knowledge, sound deletion and regular and irregular word reading.

#### Home learning environment (*t*1*, t*2)

Parents completed the HLE index (Melhuish et al., 2008) by reporting the frequency of 7 routine learning activities they do at home with their child using a 0 to 7 scale.

#### School readiness (*t*2)

The Brief Early Skills and Support Index (BESSI; https://www.cfr.cam.ac.uk/tests‐questionnaires/bessi) was completed by school staff to assess each child's school readiness at the end of nursery. BESSI is a 30‐item questionnaire which assesses how prepared children are for school using a four‐point (strongly agree to strongly disagree) scale. Lower scores reflect greater school readiness.

### The Parents and Children Together (PACT) programme

PACT is a language teaching programme designed to be delivered by parents to their children in the year before they start school (i.e., aged 3–4 years). The 30‐week programme (Burgoyne et al., 2018) supports children's early language and emergent literacy skills through 3 key components: *Shared book reading:* Parent‐child interactive book reading which uses prompts to encourage children's active participation; *Vocabulary:* Targeted teaching of vocabulary following the principles of multiple context learning (Beck et al., 2013); *Narrative skills (storytelling):* Activities to develop sequencing, summarising and storytelling skills. An overview of the teaching programme and an example of a teaching session is provided in (Burgoyne et al. (2018) Appendix C).

As in the second trial, the current trial used a revised version of the programme materials published by BookTrust (https://www.booktrust.org.uk/). These comprise six, 5‐week teaching ‘blocks’ which are linked to a theme (e.g., animals). Weeks 1–4 of each block introduce new learning material, and the final (5th) week consolidates and extends learning. Parents receive a new block of materials every 5 weeks, including books and resources for activities. Parents were asked to work on the programme with their child at home for 20 min a day, 5 days a week, for 30 weeks (i.e., 150 sessions; 50 h of teaching in total).

#### Programme training and support

Schools were asked to nominate 1 or 2 staff members as PACT Lead for the programme. This role involved recruiting and supporting families throughout the programme and acting as the main contact for the project. Prior to family recruitment, PACT Leads attended a live, online training session delivered by the research team. Training covered the PACT programme materials, recruiting and supporting families, and the background, aims, and design of the project alongside data collection methods.

Intervention group families (*N* *=* 185) were asked to attend a live, online 1.5‐2‐h researcher‐led training session prior to starting the programme. All family members who would be involved in programme delivery were invited to attend. Multiple dates/times for training were offered to provide maximum availability for families. Training covered the background and aims of the project, but focussed on the programme materials and how to deliver the programme at home, including guidance and examples of how to use the resources effectively. Parents were also given information on how to record programme delivery (see below).

In total, 145/185 (78%) intervention group families attended online researcher‐delivered training. Families unable to attend these sessions (*N* = 40) were either trained by their PACT Lead in school using the same training materials (*N* = 4, 2%) or were provided with a recording of the training and confirmed when they had watched this (*N* = 29, 16%). Some families (*N* = 7, 4%) did not confirm they had watched the recording and we are not able to confirm they received training.

To monitor programme delivery, families were asked to record when/if they delivered each session, and whether their child enjoyed it. Parents could do this via an app or using paper record forms. Eighty‐two percent (152/185) of intervention group families completed these records (it is unknown if other intervention group families completed the programme but not the records or whether they didn't complete the programme). The mean number of sessions recorded as complete by the end of the intervention period and before the immediate post‐test was 98.1/150 sessions (standard deviation = 48.6) equating to an average 19.62 weeks completed in families reporting this data.

### Statistical analysis

#### Primary outcome

The primary outcome measure was a latent language variable which combined four variables from the LanguageScreen subscales (Expressive vocabulary; Receptive vocabulary; Listening comprehension; Sentence repetition). The weightings of this latent variable were calculated through confirmatory factor analysis based on the raw scores from the LanguageScreen subscales and included a full information maximum likelihood estimation (Cham et al., 2017) to account for missing data. The use of a latent language outcome was informed by prior PACT trials (e.g., Burgoyne et al., 2018, 2024), which demonstrated that combining multiple subdomains of early language through a confirmatory factor model can yield a more reliable and theoretically grounded estimate of overall language ability. This was analysed using multilevel models (MLM) through the statistical software package RStudio (RStudio team, 2022) with the pre‐test LanguageScreen latent language variable (the baseline assessments) included as a covariate for baseline adjustment. Effect sizes and confidence intervals were computed using the standardised mean difference (Hedges' *g*) (Hedges, 1981), based on the unconditional variance of the outcome. Estimates were derived using the R package eefAnalytics, with school‐level random intercepts and random slopes for the intervention variable, which is the commonly used modelling approach for multisite trials. Intraclass correlation coefficients (ICCs) were estimated from unconditional MLM with a random intercept for school only, to quantify the proportion of variance attributable to clustering.

Analysis of outcomes followed Intention to Treat principles using MLM adjusted for prior attainment to account for variability in child attainment and the intervention effect across schools. This allowed us to test whether the estimated effects of the intervention were constant across schools.

#### Secondary outcome

Unlike the primary outcome, secondary outcomes were analysed as separate measures and not combined into latent constructs, as each represented distinct domains assessed through validated, standardised instruments. Non‐latent variable secondary outcomes (including HLE, BESSI, BPVS 3, CELF, APT Information, APT Grammar and YARC subtests of letter sound knowledge, sound deletion, and regular and irregular word reading) were analysed using MLM, with school and school‐by‐intervention added as random effects. Where available, an appropriate pre‐test variable (e.g., baseline LanguageScreen assessments) was included in the model as a covariate for baseline adjustment. Effect sizes and confidence intervals were calculated using Hedges' *g* (Hedges, 1981) based on the unconditional variance of each outcome. ICCs were also reported.

## RESULTS

At pretest (*t*1) we obtained LanguageScreen data from 365 children, of whom 339 completed the same measure at immediate posttest (i.e., overall attrition for the primary outcome measure *t*2 = 7%) and 333 completed at delayed posttest (*t*3; 9% attrition). Rates of attrition were essentially identical between groups (Figure 1).

Descriptive statistics (means and standard deviations) on all primary and secondary outcome measures at pre‐test, immediate post‐test, and delayed post‐test are reported by group in Table 1 along with intervention effect sizes (Hedges' *g* with 95% confidence intervals). Table 1 also reports unadjusted ICCs estimated from null MLM. The intervention and control groups had similar scores at pretest (LanguageScreen and HLE). Levels of missing data on secondary outcome measures at immediate and delayed post‐test varied. Standard scores (means and standard deviations) and % of children scoring <10th percentile on the LanguageScreen subtests are reported in Table 2 to contextualise the sample against population norms.

**TABLE 1:** Mean scores (SD) for intervention and control group for primary and secondary outcome measures at baseline (*t*1), immediate post‐test (*t*2), and delayed post test (*t*3) with effect sizes for intervention effects.

	PACT programme *N* = 186		Control group *N* = 186		Effect size (Hedges' *g*) [95% CI]	Unadjusted intra‐class correlation coefficient
	*N*	Mean (SD)	*N*	Mean (SD)	PACT versus control	
Age (months) (*t*1)	181	42.83 (3.41)	184	43.35 (3.41)		
Primary outcome						
LanguageScreen latent variable						
*t*1	171	−0.04 (3.62)	168	0.02 (3.87)		
*t*2	171	0.03 (2.84)	168	−0.03 (3.22)	0.03 (−0.23, 0.28)	0.111
*t*3	165	0.08 (2.32)	168	−0.08 (2.81)	0.09 (−0.14, 0.33)	0.049
Secondary outcomes						
Researcher‐delivered language tests (raw scores)						
Expressive vocabulary (CELF)						
*t*2	183	18.69 (7.15)	178	18.50 (7.06)	0.03 (−0.22, 0.29)	0.132
*t*3	175	24.06 (6.30)	173	24.39 (6.91)	−0.03 (−0.27, 0.22)	0.113
Receptive vocabulary (BPVS)						
*t*2	176	62.32 (17.06)	169	60.52 (17.44)	0.11 (−0.14, 0.36)	0.097
*t*3	177	76.26 (13.67)	173	76.61 (14.20)	−0.03 (−0.30, 0.24)	0.170
APT information						
*t*2	182	24.49 (5.76)	178	24.67 (5.85)	−0.01 (−0.26, 0.25)	0.109
*t*3	175	27.99 (5.52)	173	27.76 (4.97)	0.04 (−0.19, 0.28)	0.076
APT grammar						
*t*2	182	19.84 (5.95)	178	19.89 (6.22)	0.01 (−0.24, 0.25)	0.082
*t*3	175	22.74 (5.77)	173	23.61 (5.48)	−0.13 (−0.37, 0.11)	0.071
Home learning environment (HLE index)						
*t*1	115	29.13 (10.04)	127	29.31 (9.08)		
*t*2	116	29.00 (9.37)	119	31.02 (10.09)	−0.07 (−0.35, 0.21)	0.000
School readiness (BESSI)^a^						
Behavioural adjustment *t*2	162	1.93 (2.57)	162	2.36 (3.22)	−0.14 (−0.40, 0.13)	0.096
Language and cognition *t*2	162	0.63 (1.14)	162	0.78 (1.26)	−0.11 (−0.37, 0.15)	0.103
Daily living skills *t*2	162	0.63 (1.13)	162	0.79 (1.33)	−0.13 (−0.38, 0.12)	0.057
Family support *t*2	162	0.65 (1.21)	162	2.80 (1.31)	−0.12 (−0.40, 0.16)	0.162
Total score *t*2	162	3.86 (4.55)	162	4.75 (5.85)	−0.15 (−0.43, 0.13)	0.146
Early literacy (YARC)						
Letter sound knowledge *t*3	174	14.79 (2.38)	173	14.68 (2.41)	0.05 (−0.25, 0.36)	0.244
Sound deletion *t*3	173	4.44 (2.65)	172	4.81 (2.42)	−0.11 (−0.35, 0.14)	0.090
Regular word reading *t*3	175	9.47 (4.11)	173	9.18 (4.06)	0.12 (−0.17, 0.41)	0.218
Irregular word reading *t*3	175	2.97 (3.64)	173	2.80 (3.78)	0.06 (−0.20, 0.32)	0.121

Abbreviations: BESSI, Brief Early Skills and Support Index; BPVS, British Picture Vocabulary Scales; CELF, clinical evaluation of language fundamentals; HLE, home learning environment; YARC, York Assessment for Reading Comprehension.

^a^ Lower score = greater school readiness.

**TABLE 2:** LanguageScreen age‐standardised scores for intervention and control group at baseline (*t*1), immediate post‐test (*t*2), and delayed post test (*t*3).

	PACT programme			Control group		
	*N*	Mean (SD)	%<10th percentile	*N*	Mean (SD)	%<10th percentile
Expressive vocabulary						
*t*1	181	97.96 (13.92)	9.4%	184	97.11 (15.17)	15.2%
*t*2	174	105.87 (12.75)	7.5%	168	104.74 (15.06)	15.5%
*t*3	167	108.22 (12.11)	9.0%	168	107.18 (13.70)	11.9%
Receptive vocabulary						
*t*1	181	99.39 (14.94)	9.9%	184	97.26 (14.78)	12.0%
*t*2	174	102.75 (14.41)	10.9%	168	102.26 (14.47)	9.5%
*t*3	167	105.96 (13.64)	10.8%	168	104.90 (13.01)	12.5%
Listening comprehension						
*t* 1	181	95.93 (13.01)	23.2%	184	96.31 (13.20)	24.5%
*t*2	174	103.63 (14.54)	9.8%	168	103.90 (15.42)	11.9%
*t*3	167	107.14 (12.44)	8.4%	168	107.18 (13.50)	14.3%
Sentence repetition						
*t*1	181	97.44 (13.20)	8.3%	184	97.34 (15.08)	15.8%
*t*2	174	103.90 (14.48)	9.2%	168	103.96 (15.36)	13.1%
*t*3	167	104.91 (11.65)	8.4%	168	104.55 (12.97)	12.5%

*Note*: Age‐standardised scores are reported descriptively for the LanguageScreen subtests at each timepoint (*t*1, *t*2, *t*3) to support interpretation against population norms. These scores were not used in primary outcome modelling. Proportions scoring below the 10th percentile are calculated based on the standardised score distribution.

### Effects on primary outcome measures

**FIGURE 2:**
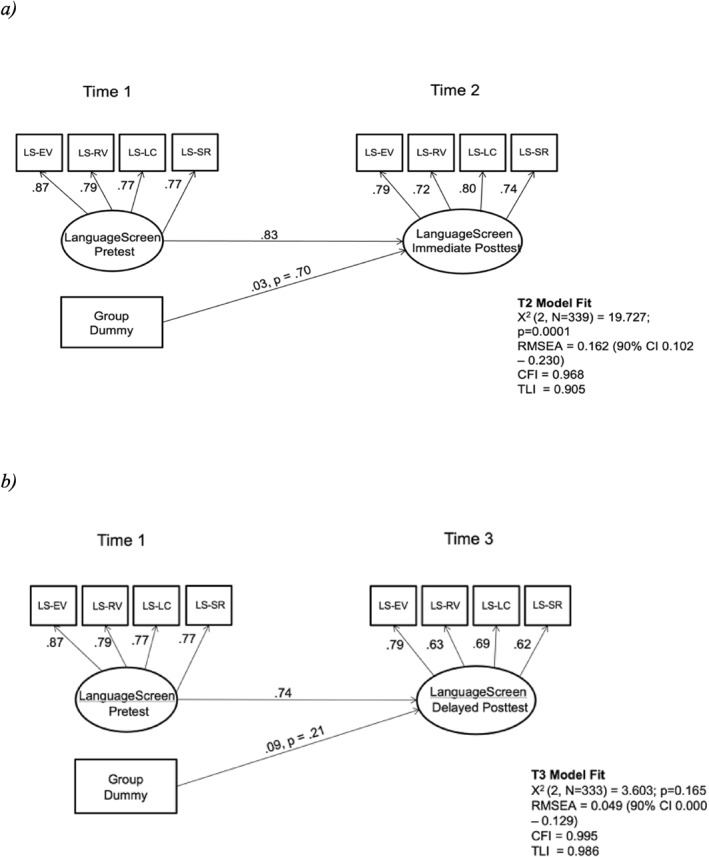
Models showing the effects of the intervention on (A) LanguageScreen at immediate post‐test (*t*2); (B) LanguageScreen at delayed post‐test (*t*3). LS‐EV, LanguageScreen Expressive Vocabulary; LS‐LC, LanguageScreen Listening Comprehension; LS‐RV, LanguageScreen Receptive Vocabulary; LS‐SR, LanguageScreen Sentence Repetition.

Our primary outcome was a language latent variable defined by the four LanguageScreen Subtests (i.e., Receptive Vocabulary, Expressive Vocabulary, Listening Comprehension and Sentence Repetition) at immediate post‐test (*t*2) and delayed post‐test (*t*3). Such a measure assesses a latent construct that uses shared variance of the language subtests and therefore captures all dimensions of the observed language variables. The models used are shown in Figure 2A,B and provide satisfactory fit to the data (immediate post‐test (*t*2): CFI = 0.968; TLI = 0.905, Root Mean Square Error of Approximation (RMSEA) = 0.162 [90% CI 0.102, 0.230]; SRMR (Standardised Root Mean Square Residual) = 0.028; delayed post‐test (*t*3): CFI = 0.995; TLI = 0.986; RMSEA = 0.049 [90% CI 0.000, 0.129]; SRMR = 0.015). Values of CFI > 0.90, TLI > 0.90, RMSEA < 0.08 and SRMR < 0.08 are considered indicative of acceptable model fit.

The most critical result is that there are no significant group differences at immediate posttest (*p* = 0.70, Hedges' *g* = 0.03 [95% CI −0.23, 0.28]) or delayed posttest (*p* = 0.21, Hedges' *g* = 0.09 [95% CI −0.14, 0.33]). The inclusion of age in the multi‐level model did not change the observed effect size from the primary analysis and inclusion of an interaction between age and intervention was not significant (*p* = 0.45) showing no difference in the effect of the programme for older compared to younger children. The ICC for the primary outcome at immediate post‐test was 0.111 and 0.049 at delayed post‐test, indicating low to moderate clustering at the school level.

**FIGURE 3:**
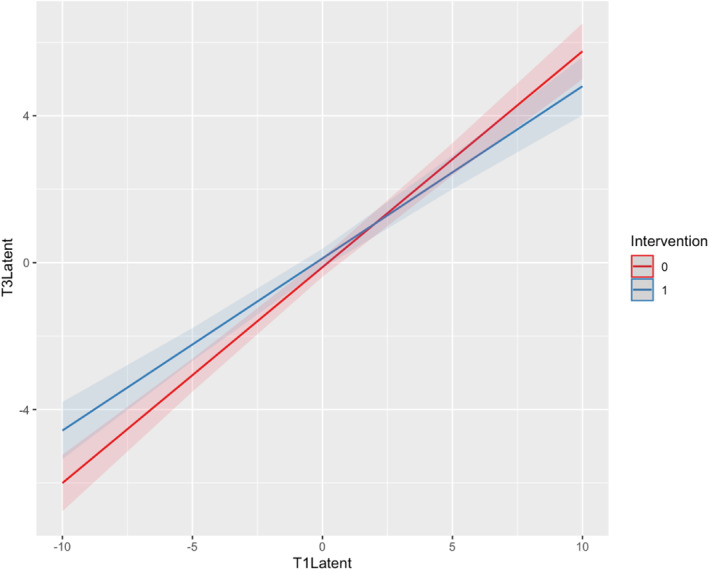
Scatterplot showing the relationship between the outcome variable (delayed post‐test language factor score) and the covariate (pre‐test language factor score) for the language programme (1) and control (0) groups.

A critical assumption for these analyses is that there are equivalent slopes between language pretest and posttest/delayed posttest factor scores across groups. Analyses which included the interaction term between the pretest factor scores and the group dummy code showed no interaction at immediate posttest confirming that the slopes relating pretest to posttest language scores do not differ between groups (*p* = 0.099). In other words, at immediate posttest children responded similarly to the programme regardless of their starting language skills. At delayed posttest, however, the interaction between group and the language pretest factor was significant (see Figure 3) and so was retained in the model (standardised difference in slopes −0.119 [95% CI −0.222, −0.017]; *p* = 0.024).

The shallower slope for the PACT programme group shows that the effect of the programme is greatest for children with the weakest language skills at pretest, while children with the best language skills at pretest show no benefit.

A Complier Average Causal Effect analysis was conducted to assess the relationship between the number of PACT sessions completed (intervention group only) and the LanguageScreen latent variable outcome at immediate and delayed posttest (see Supporting Information S1: Tables S3–S5). While there was a positive relationship between number of sessions completed and the estimated effect size at immediate and delayed posttest, at immediate posttest (*t*2) even at high levels of compliance this effect size remained small (for those completing at least 90% of PACT sessions, *n* = 52, Hedges' *g* = 0.06). However, at delayed posttest (*t*3) there was an increase in effect size for those who were more compliant (Hedges' *g* = 0.22 where more than 80% of PACT sessions were completed, *n* = 86, and Hedges' *g* = 0.33 for those completing more than 90% of sessions, *n* = 52). The point estimate is relatively large but not statistically significant, which is consistent with the smaller n and correspondingly wider CI at high ‘dose’. Nonetheless these analyses indicate that the intervention may have a delayed effect and be more beneficial for those who complete more sessions.

### Effects on secondary outcome measures

#### Researcher‐delivered language measures

Raw scores on researcher‐delivered language measures (receptive vocabulary (BPVS), expressive vocabulary (CELF), and expressive information and grammar (APT)) at immediate and delayed post‐test were used to examine potential effects of intervention on specific domains of language skill and to triangulate with the primary outcome measure (see Table 1) controlling for equivalent LanguageScreen scores at baseline (expressive and receptive vocabulary) or for the pre‐test LanguageScreen latent variable (APT information and grammar). At both immediate (*t*2) and delayed post‐test (*t*3), moderate correlations were observed between baseline measures and subsequent scores: At *t*2, the correlations were *r*(BPVS, T1RV) = 0.69, *r*(CELF, T1EV) = 0.70, APT information (*r* = 0.52), and APT grammar (*r* = 0.55). At *t*3, the correlations were slightly weaker: *r*(BPVS, T1RV) = 0.62, *r*(CELF, T1EV) = 0.69, APT information (*r* = 0.47), and APT grammar (*r* = 0.50). None of the group differences were significant and effect sizes (Hedges' *g*) in these models were small at both immediate post‐test (*t*2; BPVS *p* = 0.23, *g* = 0.11; CELF *p* = 0.67, *g* = 0.03, APT Information *p* = 0.96, *g* = −0.01, APT Grammar *p* = 0.94, *g* = 0.01) and delayed post‐test (*t*3; BPVS *p* = 0.70, *g* = −0.03; CELF *p* = 0.73, *g* = −0.03, APT Information *p* = 0.69, *g* = 0.04, APT Grammar *p* = 0.20, *g* = −0.13).

#### Early literacy skills

Group differences on measures of early literacy skills (regular and irregular word reading, letter‐sound knowledge, sound deletion) at delayed post‐test were analysed using the LanguageScreen latent variable at pre‐test as a covariate. There were moderate correlations between the pre‐test latent variable and the scores for regular (*r* = 0.47) and irregular word reading (*r* = 0.28), letter‐sound knowledge (*r* = 0.41) and sound deletion (*r* = 0.44) at delayed post‐test. No significant group differences were found: letter‐sound knowledge (*p* = 0.59, *g* = 0.05; sound deletion *p* = 0.31, *g* = −0.11; regular word reading *p* = 0.42; *g* = 0.12; irregular word reading *p* = 0.65; *g* = 0.06).

#### Home learning environment (HLE)

The HLE Index at *t*1 was used as a pre‐test score when analysing post‐test (*t*2) HLE Index as a secondary outcome; the correlation between these variables was *r* = 0.70. There was no evidence that the intervention group had higher HLE scores than the control group at immediate post‐test (*p* = 0.50; Hedges' *g* = −0.07 [95% CI −0.35, 0.21]).

#### School readiness (BESSI)

There was no baseline measure for the BESSI at delayed post‐test (*t*3). Though scores were in favour of the intervention group, there were no significant group differences (*p* = 0.19; Hedges' *g* = −0.15 [95% CI −0.43, 0.13]).

## DISCUSSION

This paper reports findings from a large‐scale, pre‐registered, RCT of a parent‐delivered early language teaching programme for preschool children (aged 3–4 years). Findings showed no significant effects on measures of early language and literacy, the HLE, or school readiness: Effect sizes on all measures were small (Hedges' *g* ranged between −0.15 and 0.12) and did not consistently favour the intervention group.

These findings build on two previous evaluations of this programme. The first trial (Burgoyne et al., 2018) showed improvements in early language (*d* = 0.21) and narrative skills (*d* = 0.36) immediately after intervention, with stronger effects on language outcomes 6‐month later (*d* = 0.34); at that point, effects were also found on measures of word reading (*d* = 0.35) and letter sound knowledge (*d* = 0.42). The second evaluation of PACT (Burgoyne et al., 2024) was impacted by COVID‐19 and as such was difficult to interpret: however, as in the current study there were no significant effects on language outcomes assessed 10 months after teaching ended (*g* = 0.01).

Though effects were not significant it is worth noting that, similar to the first trial, the current trial found stronger effects on language at delayed (Hedges' *g* = 0.09) relative to immediate post‐test (Hedges' *g* = 0.03) and some of the strongest effects on a measure of early literacy that is, regular word reading (*d* = 0.12). Though these effects are typically considered small (though note Kraft (2020) suggests an effect size of 0.09 should be considered moderate) and not significant in either case it may suggest some ‘sleeper effects’ could emerge over time. Further, at delayed post‐test, stronger effects were seen at higher dosage (Hedges' *g* = 0.22 and 0.33 for those completing more than 80% and 90% of the programme respectively) and for those with weaker language skills. These are large effect sizes, in line with those from the first trial, which suggest stronger effects over time where compliance is high. However the smaller number of participants at these levels of compliance (*n* = 86 at >80% compliance and *n* = 52 at >90% compliance) and wide confidence intervals mean these effects are not significant.

The failure to replicate evidence of effectiveness seen in the first trial calls for discussion of why this might be. Our view is that the most likely explanation for the null effect obtained here is that the sample recruited were not disadvantaged and had relatively good language abilities: As shown in Table 2, only a relatively small percentage of children (8.3%–24.5%, depending on subtest) had language concerns (i.e., scores <10th percentile) at the start of the study. Parents taking part were well‐educated (see Supporting Information S1: Table S2) with over half of households having at least one parent educated to degree level (57.49%, compared to 23.62% in the first trial). Further, 61% of families in the current trial had >50 books in the home (compared to 39% in trial 1). Many families in the comparison group reported regularly reading with their children and using dialogic reading strategies (Menzies et al., 2024). Participating children and families in this study were therefore less likely to benefit from the programme than families recruited to the first trial, as they already had resources and were providing high‐quality language input at home.

We aimed to reach disadvantaged families more in need of language support by recruiting settings in geographical areas ranking highly on indices of multiple disadvantage. However, our recruited sample included settings located in postcodes with relatively low levels of disadvantage (Supporting Information S1: Table S1). Recruitment of families within settings was challenging, with seven settings unable to recruit the minimum number of 4 families and subsequently excluded from the study. This was due at least in part to ongoing Covid restrictions at the time of recruitment which made it difficult for nurseries to build relationships with new families at the beginning of the nursery year, due to being unable to conduct home visits, parents not being allowed into the nursery setting and the wearing of masks at pick‐up and drop‐off time (Menzies et al., 2024). This led to fewer disadvantaged families taking part in the study as these families are already harder to engage (Campbell, 2011). Future research should consider ways of specifically targeting disadvantaged families and should allow time to build rapport with families before recruitment (Lingwood et al., 2020).

Our primary outcome measure in the current trial was a language latent variable defined by scores obtained on LanguageScreen, a 10‐min, computerised measure completed by school staff. LanguageScreen has demonstrated reliability and validity across a wide age range (3; 06 to 8; 11; Hulme et al., 2024) and has been reported to correlate well (*r's* ranging from *r* = 0.70 to 0.82) with other standardised language measures (CELF recalling sentences, CELF expressive vocabulary, and APT) with 3–4 year old children, suggesting it is a valid and reliable screener of language skills for this young age group (West et al., 2024). The reliance on this single, brief, educator‐administered test does however only provide a limited picture of children's language abilities and may be less sensitive to change over time. A latent variable approach which combined LanguageScreen with data from in‐depth language assessments (as in West et al., 2024) would be consistent with the way in which language was operationalised in the first trial and may have been more effective in capturing intervention effects. However, the researcher‐delivered, in‐depth language outcomes captured at immediate and delayed post‐tests here also showed small non‐significant effect sizes making this a less likely explanation for the null effects of the programme.

There are implementation challenges in the current study that are worth discussing here as they contribute to understanding the conditions under which parent‐led interventions may or may not be effective. Firstly, only 78% of families in the intervention group attended the training as intended (online, researcher‐delivered training session lasting 1.5–2 h) and it is unclear to what extent the remaining 22% of intervention group families completed training; though they were provided with a recording, web analytics suggested only a few families watched the entire recording (Menzies et al., 2024). Secondly, delivery of the programme was highly variable: Record form data collected from 82% of families (it is unclear whether the other 18% of families engaged at all with the programme) indicated that whilst some completed the 30‐week programme, others did not get started with delivery. The average completion rate from those reporting delivery was 19.6 weeks of the 30‐week programme and engagement with the programme reduced throughout delivery with only 56% of families engaging in week 20% and 43% of families engaging in week 25 (Menzies et al., 2024).

There are many factors which affect parental engagement with intervention programmes, many of which intersect with social disadvantage (Berry et al., 2023; Lingwood et al., 2020). Demands on parent time alongside other commitments (e.g., work, caring responsibilities) as well as practical constraints such as lack of transport or childcare (Hutchings et al., 2004) are strong predictors of attendance and engagement with parent programmes (Burney et al., 2024; Whittaker & Cowley, 2012). PACT programme training was intentionally relatively brief (1.5–2 h) to reduce demands on parents' time, and in the current study was held online on multiple days and at varying times of day to increase accessibility and remove practical barriers to attendance (Hutchings et al., 2004). Nonetheless, a substantial proportion of families (22%) were unable to engage. The PACT programme was also time‐intensive to deliver over a sustained period. Families reported finding time to deliver the programme challenging and when families stopped engaging with PACT, time and disrupted home circumstances were cited as key reasons (Menzies, 2024). A lack of peer support and rapport with families can also reduce motivation to engage (Boag‐Munroe & Evangelou, 2012). Leads were trained to provide support to families in the current study but in practice this was impacted by ongoing effects of COVID‐19, particularly staff illnesses and reduced face to face contact with families. As greater engagement with the programme is likely to lead to better outcomes, consideration should be made of how better to support families to sustain programme delivery with specific targeting of disadvantaged families who are already likely to have more unstable home circumstances (Cavanagh & Fomby, 2019).

Further post‐COVID contextual differences are worth noting: Following the pandemic, the national narrative in the UK highlighted negative effects on child development, particularly in the early years, and the need for children to ‘catch up’: This increased awareness and attention to early language skills and led to considerable efforts to support early language development, including the national roll‐out of school‐delivered early language intervention. Nurseries may have consequently compensated for children in the comparison group, and indeed there was some evidence that families and children in this group received more advice and support from nurseries than children in the intervention group (Menzies et al., 2024). The contextual landscape in which the current trial was situated was therefore inherently different to that of Burgoyne et al. (2018) reducing the likelihood of replicating results.

In conclusion, the current study failed to replicate previous positive effects of the parent‐delivered early language teaching programme: While nurseries and parents are positive about the programme and observe benefits for children (Menzies et al., 2024), no significant effects were found on measures of children's early language and literacy skills, HLE, or school readiness. Programmes such as that evaluated here may only be effective when targeted at children most in need, such as those from disadvantaged backgrounds and those with neurodevelopmental disorders in which language is affected (Donolato et al., 2023, though see Wake et al., 2015) though further research is needed to support this. Future research would benefit from careful targeting of children and families who may benefit from support, perhaps recruiting through PVI settings in disadvantaged areas where nursery quality in terms of communication, language and literacy has been shown to be poorer than in school nurseries serving these areas (Mathers & Smees, 2014) and/or selecting children with low language skills at pre‐test. Given the challenges with implementation identified here, working with these populations to identify programme adaptations to support delivery and effectiveness is a critical step in future research (Hartwell et al., 2025).

## AUTHOR CONTRIBUTIONS

**Kelly Burgoyne**: Conceptualization; funding acquisition; investigation; methodology; project administration; resources; supervision; writing—original draft; writing—review and editing. **Laura Boundy**: Data curation; investigation; methodology; project administration; supervision; writing—original draft; writing—review and editing. **Paivi Eerola**: Data curation; investigation; project administration; writing—original draft. **Qing Zhang**: Data curation; formal analysis; methodology; visualization; writing—original draft; writing—review and editing. **Jochen Einbeck**: Data curation; formal analysis; methodology; visualization; writing—original draft preparation; writing—review and editing. **Helen Cramman**: Conceptualization; funding acquisition; investigation; methodology; project administration. **Vic Menzies**: Conceptualization; data curation; formal analysis; funding acquisition; investigation; methodology; project administration; supervision; writing—original draft; writing—review and editing.

## CONFLICT OF INTEREST STATEMENT

The authors declare no conflicts of interest.

## ETHICAL CONSIDERATIONS

Informed consent/assent has been appropriately obtained (including parental consent for all participating children). Ethical approval was granted by Durham University Research Ethics Committee on 11th March 2021 (Reference no EDU‐2021‐02‐18T17_09_46‐jxjx34).

## Supporting information

Supporting Information S1

## Data Availability

Partial data that supports the findings (excluding data from LanguageScreen assessment due to legal restrictions on use and sharing of the data) has been submitted to the Education Endowment Foundation’s (EEF) Data Archive hosted by FFT (https://fft‐uat5‐ui.metadata.works/browser/landing%20[fft‐uat5‐ui.metadata.works]). This data will be made available through application to the Education Endowment Foundation.

## References

[jcv270064-bib-0001] Aikens, N. L. , & Barbarin, O. (2008). Socioeconomic differences in reading trajectories: The contribution of family, neighborhood, and school contexts. Journal of Educational Psychology, 100(2), 235–251. 10.1037/0022-0663.100.2.235

[jcv270064-bib-0002] Armstrong, R. , Scott, J. G. , Whitehouse, A. J. , Copland, D. A. , Mcmahon, K. L. , & Arnott, W. (2017). Late talkers and later language outcomes: Predicting the different language trajectories. International Journal of Speech Language Pathology, 19(3), 237–250. 10.1080/17549507.2017.1296191 28440674

[jcv270064-bib-0003] Ashraf, B. , Einbeck, J. , & Menzies, V. (2022). Statistical analysis plan. [PACT‐3]. Education Endowment Foundation. https://d2tic4wvo1iusb.cloudfront.net/production/documents/projects/20221006_PACT3_SAP_V3.2_On‐Website.pdf?v=1728577714

[jcv270064-bib-0004] Beck, I. L. , McKeown, M. G. , & Kucan, L. (2013). Bringing words to life: Robust vocabulary instruction. Guilford Press.

[jcv270064-bib-0005] Berry, V. , Melendez‐Torres, G. J. , Axford, N. , Axberg, U. , De Castro, B. O. , Gardner, F. , Leijten, P. , Handegård, B. H. , Hutchings, J. , Menting, A. , McGilloway, S. , & Scott, S. (2023). Does social and economic disadvantage predict lower engagement with parenting interventions? An integrative analysis using individual participant data. Prevention Science, 24(8), 1447–1458. 10.1007/s11121-022-01404-1 35870094 PMC10678811

[jcv270064-bib-0006] Boag‐Munroe, G. , & Evangelou, M. (2012). From hard to reach to how to reach: A systematic review of the literature on hard‐to‐reach families. Research Papers in Education, 27(2), 209–239. 10.1080/02671522.2010.509515

[jcv270064-bib-0007] Bowyer‐Crane, C. , Bonetti, S. , Compton, S. , Nielsen, D. , D’Apice, K. , & Tracey, L. (2021). The impact of Covid‐19 on school starters: Interim briefing 1. Parent and school concerns about children starting school. Education Endowment Foundation. https://educationendowmentfoundation.org.uk/public/files/Impact_of_Covid19_on_School_Starters_‐_Interim_Briefing_1_‐_April_2021_‐_Final.pdf

[jcv270064-bib-0008] Bowyer‐Crane, C. , Snowling, M. J. , Duff, F. J. , Fieldsend, E. , Carroll, J. M. , Miles, J. , Götz, K. , & Hulme, C. (2008). Improving early language and literacy skills: Differential effects of an oral language versus a phonology with reading intervention. Journal of Child Psychology and Psychiatry, 49(4), 422–432. 10.1111/j.1469-7610.2007.01849.x 18081756

[jcv270064-bib-0009] Burgoyne, K. , Gardner, R. , Whiteley, H. , Snowling, M. J. , & Hulme, C. (2018). Evaluation of parent‐delivered early language enrichment programme: Evidence from a randomised controlled trial. Journal of Child Psychology and Psychiatry, 59(5), 545–555. 10.1111/jcpp.12819 28940192

[jcv270064-bib-0010] Burgoyne, K. , Hargreaves, S. , Akhter, N. , Cramman, H. , Eerola, P. , Einbeck, J. , & Menzies, V. (2024). Lack of effect of a parent‐delivered early language intervention: Evidence from a randomised controlled trial completed during COVID‐19. JCPP Advances, 5(3), e12279. 10.1002/jcv2.12279 40979730 PMC12446704

[jcv270064-bib-0011] Burney, V. , McCann, C. M. , & Arnold‐Saritepe, A. (2024). Parent engagement in child‐focused interventions: A systematised review of qualitative allied health literature. Child and Youth Care Forum, 53(6), 1451–1486. 10.1007/s10566-024-09797-6

[jcv270064-bib-0012] Campbell, C. (2011). How to involve hard‐to‐reach parents: Encouraging meaningful parental involvement with schools. National College for School Leadership. https://assets.publishing.service.gov.uk/media/5a7df5d7e5274a2e8ab44eec/how‐to‐involve‐hard‐to‐reach‐parents‐summary.pdf

[jcv270064-bib-0013] Cavanagh, S. , & Fomby, P. (2019). Family instability in the lives of American children. Annual Review of Sociology, 45(1), 493–513. 10.1146/annurev-soc-073018-022633 PMC7388657 32728311

[jcv270064-bib-0014] Cham, H. , Reshetnyak, E. , Rosenfeld, B. , & Breitbart, W. (2017). Full information maximum likelihood estimation for latent variable interactions with incomplete indicators. Multivariate Behavioral Research, 52(1), 12–30. 10.1080/00273171.2016.1245600 27834491 PMC5489914

[jcv270064-bib-0015] Clegg, J. , Law, J. , Rush, R. , Peters, T. J. , & Roulstone, S. (2015). The contribution of early language development to children's emotional and behavioural functioning at 6 years: An analysis of data from the children in focus sample from the ALSPAC birth cohort. Journal of Child Psychology and Psychiatry, 56(1), 67–75. 10.1111/jcpp.12281 24980269

[jcv270064-bib-0016] Dale, P. S. , Price, T. S. , Bishop, D. V. , & Plomin, R. (2003). Outcomes of early language delay. Journal of Speech, Language, and Hearing Research, 46(3), 544–560. 10.1044/1092-4388(2003/045) 14696985

[jcv270064-bib-0017] Davies, C. , Hendry, A. , Gibson, S. P. , Gliga, T. , McGillion, M. , & Gonzalez‐Gomez, N. (2021). Early childhood education and care (ECEC) during COVID‐19 boosts growth in language and executive function. Infant and Child Development, 30(4), e2241. 10.1002/icd.2241 34220356 PMC8236989

[jcv270064-bib-0018] Donolato, E. , Toffalini, E. , Rogde, K. , Nordahl‐Hansen, A. , Lervåg, A. , Norbury, C. , & Melby‐Lervåg, M. (2023). Oral language interventions can improve language outcomes in children with neurodevelopmental disorders: A systematic review and meta‐analysis. Campbell Systematic Reviews, 19(4), e1368. 10.1002/cl2.1368 38024782 PMC10680434

[jcv270064-bib-0019] Dowdall, N. , Melendez‐Torres, G. J. , Murray, L. , Gardner, F. , Hartford, L. , & Cooper, P. J. (2020). Shared picture book reading interventions for child language development: A systematic review and meta‐analysis. Child Development, 91(2), e383–e399. 10.1111/cdev.13225 30737957

[jcv270064-bib-0020] Dunn, L. , & Dunn, D. , & National Foundation for Educational Research . (2009). The British picture vocabulary scale (3rd ed.). GL Assessment. https://www.gl‐assessment.co.uk/assessments/products/british‐picture‐vocabulary‐scale/

[jcv270064-bib-0021] Eadie, P. , Bavin, E. L. , Bretherton, L. , Cook, F. , Gold, L. , Mensah, F. , Wake, M. , & Reilly, S. (2021). Predictors in infancy for language and academic outcomes at 11 years. Pediatrics, 147(2), e20201712. 10.1542/peds.2020-1712 33431588

[jcv270064-bib-0022] Fitzpatrick, C. , Boers, E. , & Pagani, L. S. (2020). Kindergarten readiness, later health, and social costs. Pediatrics, 146(6), e20200978. 10.1542/peds.2020-0978 33139455

[jcv270064-bib-0023] Fricke, S. , Burgoyne, K. , Bowyer‐Crane, C. , Kyriacou, M. , Zosimidou, A. , Maxwell, L. , Lervåg, A. , Snowling, M. J. , & Hulme, C. (2017). The efficacy of early language intervention in mainstream school settings: A randomized controlled trial. Journal of Child Psychology and Psychiatry, 58(10), 1141–1151. 10.1111/jcpp.12737 28524257

[jcv270064-bib-0024] Hartwell, K. , Pagnamenta, E. , Stojanovik, V. , Baxter, R. , Burgoyne, K. , & Fletcher, A. L. (2025). The power of partnership: Adapting early language intervention for children with down syndrome through family‐researcher collaboration. International Journal of Language and Communication Disorders, 60(6), e70139. 10.1111/1460-6984.70139 41076552 PMC12515274

[jcv270064-bib-0025] Hedges, L. V. (1981). Distribution theory for glass's estimator of effect size and related estimators. Journal of Educational Statistics, 6(2), 107–128. 10.3102/10769986006002107

[jcv270064-bib-0026] Hulme, C. , McGrane, J. , Duta, M. , West, G. , Cripps, D. , Dasgupta, A. , Hearne, S. , Gardner, R. , & Snowling, M. (2024). LanguageScreen: The development, validation, and standardization of an automated language assessment app. Language, Speech, and Hearing Services in Schools, 55(3), 904–917. 10.1044/2024_lshss-24-00004 38776269

[jcv270064-bib-0027] Hulme, C. , Nash, H. M. , Gooch, D. , Lervåg, A. , & Snowling, M. J. (2015). The foundations of literacy development in children at familial risk of dyslexia. Psychological Science, 26(12), 1877–1886. 10.1177/0956797615603702 26525072 PMC4676358

[jcv270064-bib-0028] Hulme, C. , Stothard, S. E. , Clarke, P. , Bowyer‐Crane, C. , Harrington, A. , Truelove, E. , & Snowling, M. J. (2009). York assessment of reading for comprehension: Early reading. GL Assessment.

[jcv270064-bib-0029] Hutchings, J. , & Webster‐Stratton, C. (2004). Community‐based support for parents. In Handbook of parenting: Theory and research for practice (pp. 334–351).

[jcv270064-bib-0030] Kraft, M. A. (2020). Interpreting effect sizes of education interventions. Educational Researcher, 49(4), 241–253. 10.3102/0013189x20912798

[jcv270064-bib-0031] Leech, K. A. , McNally, S. , Daly, M. , & Corriveau, K. H. (2022). Unique effects of book‐reading at 9‐months on vocabulary development at 36‐months: Insights from a nationally representative sample of Irish families. Early Childhood Research Quarterly, 58, 242–253. 10.1016/j.ecresq.2021.09.009

[jcv270064-bib-0032] Le Roux, A. (2012). The production and use of wordless picture books in parent‐child reading: An exploratory study within a South African context. Stellenbosch University Book‐Sharing Meta‐Analysis.e397.

[jcv270064-bib-0033] Lingwood, J. , Levy, R. , Billington, J. , & Rowland, C. (2020). Barriers and solutions to participation in family‐based education interventions. International Journal of Social Research Methodology, 23(2), 185–198. 10.1080/13645579.2019.1645377

[jcv270064-bib-0034] Marmot, M. (2020). Health equity in England: The marmot review 10 years on. BMJ, 368, m693. 10.1136/bmj.m693 32094110

[jcv270064-bib-0035] Mathers, S. , & Smees, R. (2014). Quality and inequality. Do three‐ and four year olds in deprived areas experience lower quality early years provisions? Nuffield Foundation. https://www.nuffieldfoundation.org/sites/default/files/files/Quality_inequality_childcare_mathers_29_05_14.pdf

[jcv270064-bib-0036] McKean, C. , Mensah, F. , Eadie, P. , Bavin, E. , Bretherton, L. , Cini, E. , & Reilly, S. (2015). Levers for language growth: Characteristics and predictors of language trajectories between 4 and 7 years. PLoS One, 10(8), e0134251. 10.1371/journal.pone.0134251 26241892 PMC4524638

[jcv270064-bib-0037] Melhuish, E. , Quinn, L. , Sylva, K. , Sammons, P. , Siraj‐Blatchford, I. , & Taggart, B. (2013). Preschool affects longer term literacy and numeracy: Results from a general population longitudinal study in Northern Ireland. School Effectiveness and School Improvement, 24(2), 234–250. 10.1080/09243453.2012.749796

[jcv270064-bib-0038] Melhuish, E. C. , Sylva, K. , Sammons, P. , Siraj‐Blatchford, I. , Taggart, B. , & Phan, M. (2008). Effects of the home learning environment and preschool center experience upon literacy and numeracy development in early primary school. Journal of Social Issues, 64(1), 157–188. 10.1111/j.1540-4560.2008.00550.x

[jcv270064-bib-0039] Menzies, V. , Eerola, P. , Cramman, H. , Ashraf, B. , Einbeck, J. , & Gray, H. (2022). Evaluating the impact of the parents and children together (PACT) programme on the language skills of 3‐ to 4‐year‐old nursery children, a two armed randomised controlled trial [PACT‐3]. Evaluation protocol. Education Endowment Foundation. https://d2tic4wvo1iusb.cloudfront.net/production/documents/projects/PACT_20220615_PACT3_protocol_v3.2_OnWebsite.pdf?v=1728577714

[jcv270064-bib-0040] Menzies, V. , Eerola, P.‐S. , Zhang, Q. , Cramman, H. , & Einbeck, J. (2024). Evaluating the impact of the parents and children together (PACT) programme on the language skills of 3‐ to 4‐year‐old nursery children A two‐armed randomised trial. https://educationendowmentfoundation.org.uk/

[jcv270064-bib-0041] Ministry of Housing, Communities and Local Government . (2019). English indices of deprivation 2019. GOV.UK.

[jcv270064-bib-0042] Mol, S. E. , Bus, A. G. , De Jong, M. T. , & Smeets, D. J. (2008). Added value of dialogic parent–child book readings: A meta‐analysis. Early Education & Development, 19(1), 7–26. 10.1080/10409280701838603

[jcv270064-bib-0043] Murray, A. , & Egan, S. M. (2014). Does reading to infants benefit their cognitive development at 9‐months‐old? An investigation using a large birth cohort survey. Child Language Teaching and Therapy, 30(3), 303–315. 10.1177/0265659013513813

[jcv270064-bib-0044] Murray, L. , Jennings, S. , Perry, H. , Andrews, M. , De Wilde, K. , Newell, A. , Mortimer, A. , Phillips, E. , Liu, X. , Hughes, C. , Melhuish, E. , De Pascalis, L. , Dishington, C. , Duncan, J. , & Cooper, P. J. (2023). Effects of training parents in dialogic book‐sharing: The early‐years provision in children's centers (EPICC) study. Early Childhood Research Quarterly, 62, 1–16. 10.1016/j.ecresq.2022.07.008

[jcv270064-bib-0045] Niklas, F. , & Schneider, W. (2013). Home literacy environment and the beginning of reading and spelling. Contemporary Educational Psychology, 38(1), 40–50. 10.1016/j.cedpsych.2012.10.001

[jcv270064-bib-0046] Noble, C. , Sala, G. , Peter, M. , Lingwood, J. , Rowland, C. , Gobet, F. , & Pine, J. (2019). The impact of shared book reading on children’s language skills: A meta‐analysis. Educational Research Review, 28, 100290. 10.1016/j.edurev.2019.100290

[jcv270064-bib-0047] Purpura, D. J. , Napoli, A. R. , Wehrspann, E. A. , & Gold, Z. S. (2017). Causal connections between mathematical language and mathematical knowledge: A dialogic reading intervention. Journal of Research on Educational Effectiveness, 10(1), 116–137. 10.1080/19345747.2016.1204639

[jcv270064-bib-0048] Renfrew, C. (2010). Action picture test. Speechmark Publishing.

[jcv270064-bib-0049] Roulstone, S. , Law, J. , Rush, R. , Clegg, J. , & Peters, T. (2011). Investigating the role of language in children's early educational outcomes. UK Department of Education.

[jcv270064-bib-0050] Sammons, P. , Sylva, K. , Hall, J. , Evangelou, M. , & Smees, R. (2023). Challenges facing interventions to promote equity in the early years: Exploring the ‘impact’, legacy and lessons learned from a national evaluation of children’s centres in England. Oxford Review of Education, 49(1), 114–135. 10.1080/03054985.2022.2125371

[jcv270064-bib-0051] Sammons, P. , Toth, K. , Sylva, K. , Melhuish, E. , Siraj, I. , & Taggart, B. (2015). The long‐term role of the home learning environment in shaping students’ academic attainment in secondary school. Journal of Children's Services, 10(3), 189–201. 10.1108/jcs-02-2015-0007

[jcv270064-bib-0052] Semel, E. , Wiig, E. , & Secord, W. (2006). Child evaluation of language fundamentals‐preschool UK (2nd ed.). Pearson Assessment.

[jcv270064-bib-0053] Sloat, E. A. , Letourneau, N. L. , Joschko, J. R. , Schryer, E. A. , & Colpitts, J. E. (2015). Parent‐mediated reading interventions with children up to four years old: A systematic review. Issues in Comprehensive Pediatric Nursing, 38(1), 39–56. 10.3109/01460862.2014.983279 25533602

[jcv270064-bib-0054] Wake, M. , Levickis, P. , Tobin, S. , Gold, L. , Ukoumunne, O. C. , Goldfeld, S. , Reilly, S. , Le, H. N. , & Law, J. (2015). Two‐year outcomes of a population‐based intervention for preschool language delay: An RCT. Pediatrics, 136(4), e838–e847. 10.1542/peds.2015-1337 26347428

[jcv270064-bib-0055] West, G. , Lervåg, A. , Birchenough, J. M. , Korell, C. , Rios Diaz, M. , Duta, M. , Hulme, C. , Gardner, R. , & Fairhurst, C. (2024). Oral language enrichment in preschool improves children's language skills: A cluster randomised controlled trial. Journal of Child Psychology and Psychiatry, 65(8), 1087–1097. 10.1111/jcpp.13947 38262448

[jcv270064-bib-0056] Whittaker, K. A. , & Cowley, S. (2012). An effective programme is not enough: A review of factors associated with poor attendance and engagement with parenting support programmes. Children and Society, 26(2), 138–149. 10.1111/j.1099-0860.2010.00333.x

