# Supplementary material for: Failure to replicate effects of parent‐delivered early language intervention: Evidence from a randomised controlled trial with implications for universal language intervention
Source: JCPP Adv. 2025 Nov 6:e70064. Online ahead of print. doi: 10.1002/jcv2.70064 (PMC13337129; doi:10.1002/jcv2.70064)
Supplement: Supplementary file 1 — Supporting Information S1 [file JCV2-9999-e70064-s001.docx]

**Failure to Replicate Effects of Parent-Delivered Early Language Intervention: Evidence from a Randomized Controlled Trial with Implications for Universal Language Intervention**

**Supporting Information**

Table S1 *Setting characteristics* (*N*=43)

| School-level  (categorical) | National-level mean |  | |
| --- | --- | --- | --- |
|  |  |  | Count (%) |
| Ofsted rating |  | Good  Outstanding  Requires improvement  No data available | 33  7  2  1 |
| School type |  | Community school  Voluntary aided school  Academy converter  Voluntary controlled school  Academy sponsor led  Foundation school | 17  12  5  4  4  1 |
| Location |  | Urban city and town  Urban major conurbation  Rural hamlet and isolated dwellings  Rural town and fringe  Rural village | 20  19  2  1  1 |
| Deprivation* |  | Lowest 3 deciles (highest deprivation)  Middle 3 deciles  Highest 4 deciles (least deprivation) | 23  9  11 |
| KS2 Reading progress score | 10%  9%  64%  7%  10%  - | Well below average  Below average  Average  Above average  Well above average  Missing data | 3 (7.0%)  4 (9.3%)  28 (65.1%)  5 (11.6%)  2 (4.7%)  1 (2.3%) |
| School-level  (continuous) |  |  | Mean (SD) |
| FSM eligibility | 21.6%^[[1]](#footnote-1)^. |  | 30.07% |

Note: KS2 = Key Stage 2; FSM = Free School Meals

National average at January 2022 for state-funded primary schools from (<https://explore-education-statistics.service.gov.uk/find-statistics/school-pupils-and-their-characteristics>**)**

***Based on Indices of Multiple Deprivation**

Table S2 *Sample characteristics (parent questionnaire data)*

| **Question** | **Response** | **Frequency (% of total number of responses)** | | |
| --- | --- | --- | --- | --- |
|  |  | **Total Sample** | **Intervention Group** | **Control Group** |
| Highest qualification in the household | No Formal Qualifications | 5/247 (2.02%) | 3/119 (2.52%) | 2/128 (1.56%) |
|  | GCSE or equivalent | 37/247 (14.98%) | 12/119 (10.08%) | 25/128 (19.53%) |
|  | Apprenticeship | 6/247 (2.43%) | 4/119 (3.36%) | 2/128 (1.56%) |
|  | Vocational Qualification | 23/247 (9.31%) | 11/119 (9.24%) | 12/128 (9.38%) |
|  | A-Level or equivalent | 34/247 (13.77%) | 18/119 (15.12%) | 16/128 (12.50%) |
|  | Higher Education degree or above | 142/247 (57.49%) | 71/119 (59.66%) | 71/128 (55.47%) |
|  | Missing Data |  | 66 | 57 |
| Number of children’s books at home | 0-10 | 16/248 (6.45%) | 7/120 (5.83%) | 9/128 (7.03%) |
|  | 11-20 | 18/248 (7.26%) | 8/120 (6.67%) | 10/128 (7.81%) |
|  | 21-50 | 62/248 (25.00%) | 28/120 (23.33%) | 34/128 (26.56%) |
|  | 51-100 | 76/248 (30.65%) | 38/120 (31.67%) | 38/128 (29.69%) |
|  | 100+ | 76/248 (30.65%) | 39/120 (32.50%) | 37/128 (28.91%) |
|  | Missing Data |  | 65 | 57 |

Table S3 *Compliance distribution* *(n, %) across the intervention period*

| Compliance level (%) | n (%) |
| --- | --- |
| < 10 | 23 (12.43) |
| 10 - 19 | 9 (4.86) |
| 20 - 29 | 10 (5.40) |
| 30 - 39 | 17 (9.18) |
| 40 - 49 | 13 (7.02) |
| 50 - 59 | 9 (4.86) |
| 60 - 69 | 8 (4.32) |
| 70 - 79 | 10 (4.40) |
| 80 - 89 | 34 (18.37) |
| 90 - 99 | 20 (10.81) |
| 100 | 32 (17.29) |
| Sum | 185 (100.00) |

Table S4 *Complier Average Causal Effect results for pupils in intervention group at immediate post-test (T2)*

| Compliance level | CACE (confidence intervals) |
| --- | --- |
| P >0 | 0.01 (-0.09, 0.11) |
| P >10 | 0.01 (-0.10, 0.11) |
| P >20 | 0.01 (-0.09, 0.10) |
| P >30 | 0.01 (-0.08, 0.12) |
| P >40 | 0.02 (-0.10, 0.20) |
| P >50 | 0.03 (-0.11, 0.21) |
| P >60 | 0.04 (-0.20, 0.21) |
| P >70 | 0.05 (-0.21, 0.30) |
| P >80 | 0.05 (-0.21, 0.40) |
| P >90 | 0.06 (-0.32, 0.51) |

Table S5*: Complier Average Causal Effect results for pupils in intervention group at delayed post-test (T3)*

| Compliance level | CACE (confidence intervals) |
| --- | --- |
| P >0 | 0.11 (-0.05, 0.26) |
| P >10 | 0.11 (-0.05, 0.26) |
| P >20 | 0.13 (-0.06, 0.31) |
| P >30 | 0.13 (-0.06, 0.32) |
| P >40 | 0.14 (-0.07, 0.34) |
| P >50 | 0.15 (-0.07, 0.37) |
| P >60 | 0.17 (-0.08, 0.41) |
| P >70 | 0.19 (-0.09, 0.46) |
| P >80 | 0.22 (-0.10, 0.54) |
| P >90 | 0.33 (-0.15, 0.84) |

1. National average at January 2022 for state-funded primary schools from (<https://explore-education-statistics.service.gov.uk/find-statistics/school-pupils-and-their-characteristics>**)** [↑](#footnote-ref-1)
